# Brief, weekly magnetic muscle therapy improves mobility and lean body mass in older adults: a Southeast Asia community case study

**DOI:** 10.18632/aging.204597

**Published:** 2023-03-19

**Authors:** Sharanya Venugobal, Yee Kit Tai, Jorming Goh, Sean Teh, Craig Wong, Ivan Goh, Andrea B. Maier, Brian K. Kennedy, Alfredo Franco-Obregón

**Affiliations:** 1 QuantumTx, Alexandra Hospital, Block 29 Level 1 Centre for Innovation in Healthcare, Co-Working Space, Singapore; 2 Department of Surgery, Yong Loo Lin School of Medicine, National University of Singapore, Singapore; 3 Institute of Health Technology and Innovation (iHealthtech), National University of Singapore, Singapore; 4 Biolonic Currents Electromagnetic Pulsing Systems Laboratory (BICEPS), National University of Singapore, Singapore; 5 NUS Centre for Cancer Research, Yong Loo Lin School of Medicine, National University of Singapore, Singapore; 6 Department of Physiology, Yong Loo Lin School of Medicine, National University of Singapore, Singapore; 7 Healthy Longevity Translational Research Programme, Yong Loo Lin School of Medicine, National University of Singapore, Singapore; 8 Centre for Healthy Longevity, National University Health System, Singapore; 9 Singapore Institute of Clinical Sciences, A*STAR, Singapore; 10 Department of Human Movement Sciences, @AgeAmsterdam, Faculty of Behavioural and Movement Sciences, Amsterdam Movement Sciences, Vrije Universiteit, Amsterdam, The Netherlands; 11 Department of Biochemistry, Yong Loo Lin School of Medicine, National University of Singapore, Singapore; 12 Nanomedicine Translational Research Programme, Centre for NanoMedicine, Yong Loo Lin School of Medicine, National University of Singapore, Singapore

**Keywords:** sarcopenia, intra-abdominal fat, frailty, muscle weakness, type 2 diabetes mellitus

## Abstract

Brief (10 min) weekly exposure to low energy pulsed electromagnetic fields (PEMFs) has been shown to improve human muscle mitochondrial bioenergetics and attenuate systemic lipotoxicity following anterior cruciate ligament surgical reconstruction. Here we present data generated from 101 participants, 62% female, aged 38–91 years, recruited from the QuantumTx Demo Centre in Singapore, wherein 87% of participants (*n* = 88) presented with pre-existing mobility dysfunction and 13% (*n* = 13) were healthy volunteers. Participants were recruited if: (i) not pregnant; (ii) above 35 years of age and; (iii) without surgical implants. All participants completed mobility testing, pre- and post- PEMF intervention for 12 weeks, whereas bioelectrical impedance analysis was conducted in a subgroup of 42 and 33 participants at weeks 4 and 8, respectively. Weekly PEMF exposure was associated with significant improvements in mobility (Timed Up and Go, 5 times Sit-to-Stand, and 4m Normal Gait Speed) and body composition (increased skeletal muscle mass and reduced total and visceral fat mass), particularly in the older participants. Perception of pain was also significantly reduced. PEMF therapy may provide a manner to counteract age-associated mobility and metabolic disruptions and merits future investigation in randomized controlled trials to elucidate its clinical benefits in the frail and older adult populations.

## INTRODUCTION

Muscle is our largest tissue mass and plays a major role in establishing human healthspan and lifespan [1–3]. Sarcopenia is an age-related disease characterized by reduced skeletal muscle mass, strength and physical function [4, 5]. In accordance with muscle’s imperative role in establishing organismal health and longevity, sarcopenia is associated with low quality of life [3, 6], falls, fractures [7] and mortality [8]. While regular exercise training and dietary protein supplementation are accepted efficacious means to retain muscle quality with age, they are difficult to implement, particularly in the frail and older adults. There is hence a growing need for the development of non-pharmacological and minimally invasive means to counteract sarcopenic muscle loss. Pulsed electromagnetic fields (PEMFs) may represent one such approach. PEMF-based therapies have been previously employed for clinical rehabilitation involving bone and other soft-tissue injuries, whether from acute trauma or degenerative conditions, in pre-clinical animal models [9] and humans [10]. For instance, rotator cuff tendinitis had been clinically treated with PEMFs as early as the 1980s [11]. In fact, in 1979, the United States Food and Drug Administration approved PEMFs therapy as safe and efficacious for treating non-union bone fractures within the categories of bone growth and osteogenic stimulation, opening the way for PEMF therapies to be used in the area of rehabilitation for orthopedic injuries [12, 13]. As such, rehabilitative PEMF studies have traditionally focussed on bone and connective tissue damage, as well as pain management [12–16]. Unfortunately, the translation of PEMF-based therapies for muscle maintenance has gone largely unexplored.

Since the 1970s a multitude of pre-clinical and clinical studies examining the effects of PEMF stimulation have appeared utilizing a broad range of repetition frequencies (Hz to MHz range), amplitudes (μT to 100s mT), signal gradients and symmetries, exposure durations (minutes to weeks), as well as cellular environments [12–17]. Not unexpectedly, the clinically related outcomes reported from these studies varied widely. As an illustrative example, an early double-blinded, randomized controlled trial (RCT) investigated the effects of PEMF treatment applied between 5–9 hours per day on patients with rotator cuff tendinopathy [11]. The administered PEMFs were set at an amplitude of ~3 mT at a repetition frequency of ~73 Hz. Patients receiving the magnetic field treatment reported less pain after 4 weeks relative to the sham treatment cohort. In another RCT also treating patients with rotator cuff tendinopathy [18], the PEMFs were administered at a frequency of 3 Hz, 80 mT for 20 minutes a day, twice a week for a total of 8 sessions. Although pain and shoulder function were significantly improved, the patients in this second study were also administered extracorporeal shockwave therapy, which may have partially obscured the beneficial contributions of the PEMF therapy per se. Systematic reviews have also recently highlighted that distinct tissue types likely respond distinctly to different PEMF exposure parameters [19–21]. Finally, many *in vitro* studies cannot be adequately translated to the *in vivo* scenario due to the common use of the aminoglycoside antibiotics during cell culturing. The aminoglycoside antibiotics, such as streptomycin, have been shown to attenuate the sensitivity of cells to magnetic field exposure, negating the relevancy of the determined *in vitro* field parameters to the *in vivo* condition [22, 23]. A need thus exists for greater standardization of the PEMF regimens used for specified clinical applications.

Recently, a muscle-specific, low energy (1.5 mT, 15 Hz, 10 minutes/week), PEMF paradigm has been developed and demonstrated effective in promoting muscle regeneration in cells [22, 23], animals [24] and humans [25] by virtue of its capacity to activate mitochondrial respiration [26]. By strategically targeting the leg musculature of humans with the coil system [25], this PEMF paradigm was capable of recapitulating some of the same mitochondrial-dependent regenerative and metabolic cascades typically invoked by exercise, particularly with regards to improving muscle mitochondrial bioenergetics and in attenuating systemic lipotoxicity [25]. The muscle mitochondrial network also exhibits heightened sensitivity to our magnetic stimulation paradigm [22] compared to that of other collateral tissues or progenitor cell classes [24, 27, 28] and hence is a favorable target with which to invoke systemic homeostasis for clinical exploitation. In this community case study, we investigated the capacity of this specific PEMF paradigm to improve physical functioning and increase lean mass in a Southeast Asian adult population. We provide evidence that weekly exposure of the quadriceps musculature to weak pulsing magnetic fields for 10 minutes each week, over the course of 8–12 weeks, improved physical performance as well as reduced both total (−3.9%) and visceral fat reserves (−3.7%), particularly in older participants. Interestingly, perceptions of pain were also reduced after 3 months of PEMF intervention.

## RESULTS

Cohort characteristics are provided in Table 1. The results for the Timed up and go (TUG), 5 times Sit-to-Stand (5xSTS), and 4m normal gait speed (4mNGS) mobility tests are provided in Table 2. Statistically significant improvements in functional mobility following PEMF therapy were observed for all three tests, across all age groups. Specifically, in the TUG test, 12 weeks of PEMF therapy was associated with a significant reduction in mean time of execution for the entire cohort from a pre-PEMF value of 11.25 seconds to 9.31 seconds. The older participants showed the strongest improvements from 10.77 to 8.79 seconds and from 15.35 to 13.14 seconds for ages 66–75 and 76–91 years, respectively. Given that older adults above the age of 65 who score more than 14 seconds for TUG are associated with a higher risk of falls [29], this data assumes potential clinical importance. For 5xSTS, PEMF therapy was associated with reduced times of execution from 12.71 seconds to 10.40 seconds for the entire cohort. Again, improvements were greatest for the older participants, ranging from 12.99 to 10.32 seconds and from 15.21 to 12.74 seconds for ages 66–75 and 76–91 years, respectively, faster than the predictive 15 seconds associated with a greater risk of recurrent falls in older adults [30]. 4mNGS scores generally increased following PEMF therapy, from speeds of 0.9 ms^−1^ to 1.12 ms^−1^. Specifically, the oldest group of participants (ages 76–91 years) increased in gait speed from 0.79 to 0.92 ms^−1^. Importantly, a 4mNGS speed of below 0.8 ms^−1^ is associated with increased risks for adverse health outcomes, including disability, cognitive impairment, falls, and mortality [31]. Therefore, based on these previously published studies, the changes in functional mobility reported here following 12 weeks of PEMF therapy are clinically relevant and counteract age-related trends.

**Table 1:** Study demographics.

**Study demographics**		**Total subjects**	**Total mean age (±SD)**	**Total males**	**Male mean age (±SD)**	**Total females**	**Female mean age (±SD)**
**Age (year)**	**(All)**	101	68 ± 11	38	70 ± 9	63	67 ± 12
	**(35–55)**	14	50 ± 5	2	47 ± 12	12	50 ± 4
	**(56–65)**	24	61 ± 3	8	61 ± 3	16	61 ± 3
	**(66–75)**	41	71 ± 3	18	71 ± 3	23	72 ± 2
	**(76–91)**	22	82 ± 5	10	80 ± 5	12	84 ± 5

**Table 2:** Raw values representing the mean (ave) and standard error of the mean (SEM), and mean difference (mean diff) for TUG (in secs), 5xSTS (in secs) and 4mNGS (in meters) pre- and post-PEMF therapy for 12 weeks, stratified according to age (in years).

**Mobility tests**	**Age (Year)**	**Pre (Baseline) Mean (±SD)**	**Post (PEMF Therapy) Mean (±SD)**	**Mean difference**	***p*-value**
**TUG (sec)**	[All]	11.25 ± 7.60	9.31 ± 6.20	−1.94	<0.0001
	(35–55)	8.64 ± 4.93	6.68 ± 1.87	−1.96	0.0052
	(56–65)	9.82 ± 9.75	8.24 ± 7	−1.58	0.0001
	(66–75)	10.77 ± 5.72	8.79 ± 4.74	−1.98	<0.0001
	(76–91)	15.35 ± 8.25	13.14 ± 7.87	−2.21	0.0003
**5xSTS (sec)**	(All)	12.71 ± 6.54	10.40 ± 4.77	−2.31	<0.0001
	(35–55)	10.18 ± 6.54	8.28 ± 2.59	−1.90	0.0256
	(56–65)	11.43 ± 4.94	9.64 ± 3.92	−1.79	0.0039
	(66–75)	12.99 ± 7.58	10.32 ± 4.82	−2.67	<0.0001
	(76–91)	15.21 ± 5.31	12.74 ± 5.79	−2.47	0.0115
**4mNGS (s^−1^)**	(All)	0.98 ± 0.31	1.12 ± 0.30	0.14	<0.0001
	(35–55)	1.07 ± 0.29	1.32 ± 0.24	0.25	0.0006
	(56–65)	1.08 ± 0.34	1.17 ± 0.29	0.09	0.0176
	(66–75)	1.00 ± 0.26	1.12 ± 0.27	0.13	<0.0001
	(76–91)	0.790 ± 0.31	0.92 ± 0.3	0.13	0.0002

**Figure 1:**
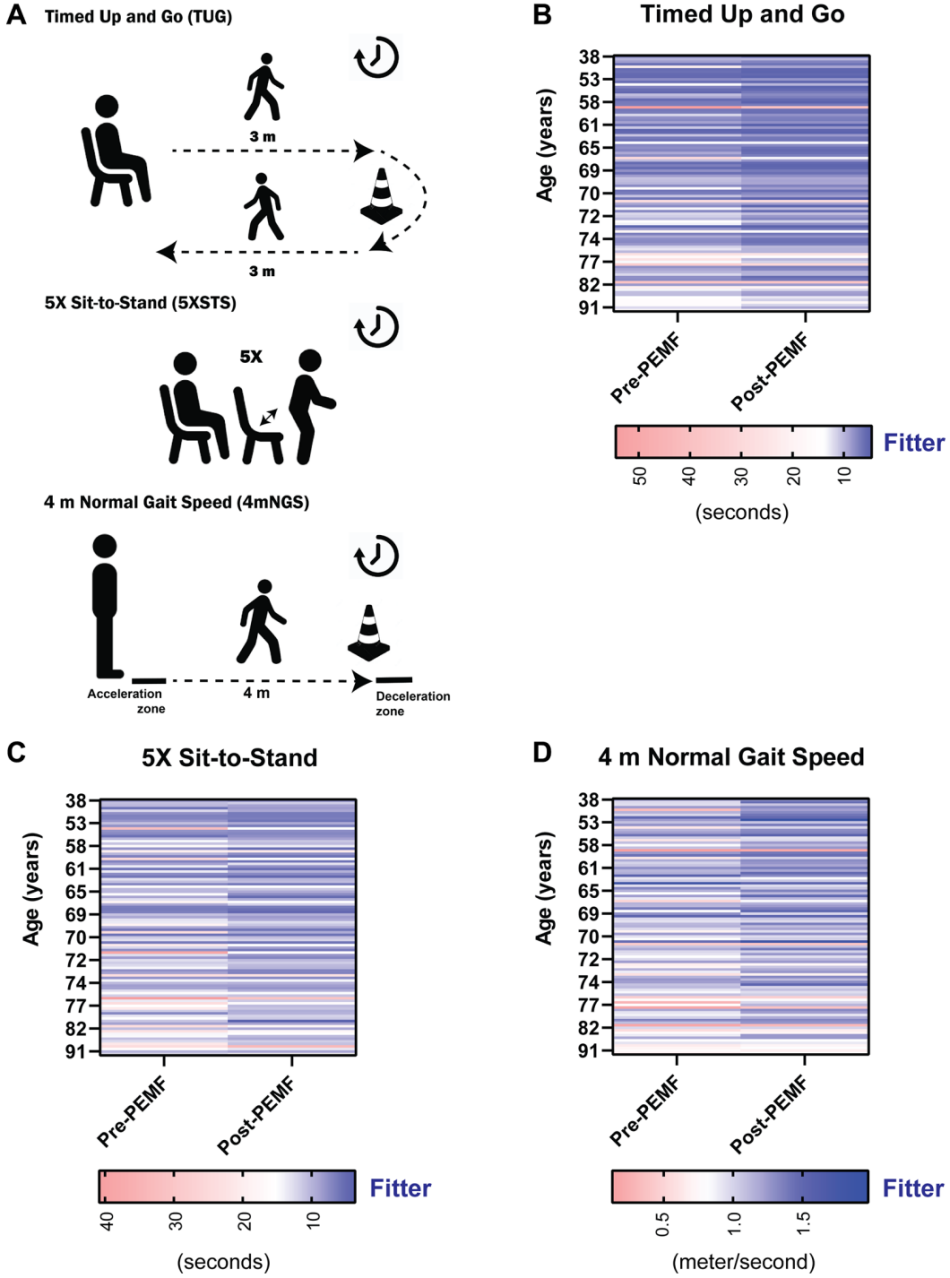
**Individual functional assessments pre- and post-PEMF therapy.** (**A**) Functional tests administered to study participants. Subject performance in the (**B**) timed-up and go (TUG; seconds), (**C**) 5X Sit to Stand (5xSTS; seconds), and (**D**) 4 m normal gait speed (4mNGS; meter/second), measured at baseline (pre-PEMF, week 1) and at week 12 (post-PEMF). Heat maps showing the responses of subjects by color gradient, with darker blue indicating functional improvement, white being the cutoffs based on known consensus for older adults, and red showing a less fit or frail characteristics. A TUG and 5xSTS score of ≥14 and ≥15 seconds, respectively are associated with increased falling risk in older adult [29, 30]. A gait speed of ≤ 0.8 m/s is correlated with an increased risk of adverse health outcomes in the older adults [31]. Statistical analysis was carried out using the Wilcoxon matched-pairs signed rank test and showed significant improvement in mobility function (*p* < 0.0001) with magnetic therapy for all three tests.

Figure 1 is a heatmap depiction of the individual responses for the entire cohort for the TUG, 5xSTS, and 4mNGS tests at baseline (pre-PEMF) and after 12 weeks of PEMF therapy. A trend was visible for each of the functional sets, whereby the pre-PEMF values worsen with greater age (increasingly redder nearer the bottom (older)), illustrating a trend of loss of function with age. In most cases, mobility and functional capacity were improved following 12 weeks of PEMF therapy (increasingly bluer on the right (post-PEMF)), irrespective of age.

**Figure 2:**
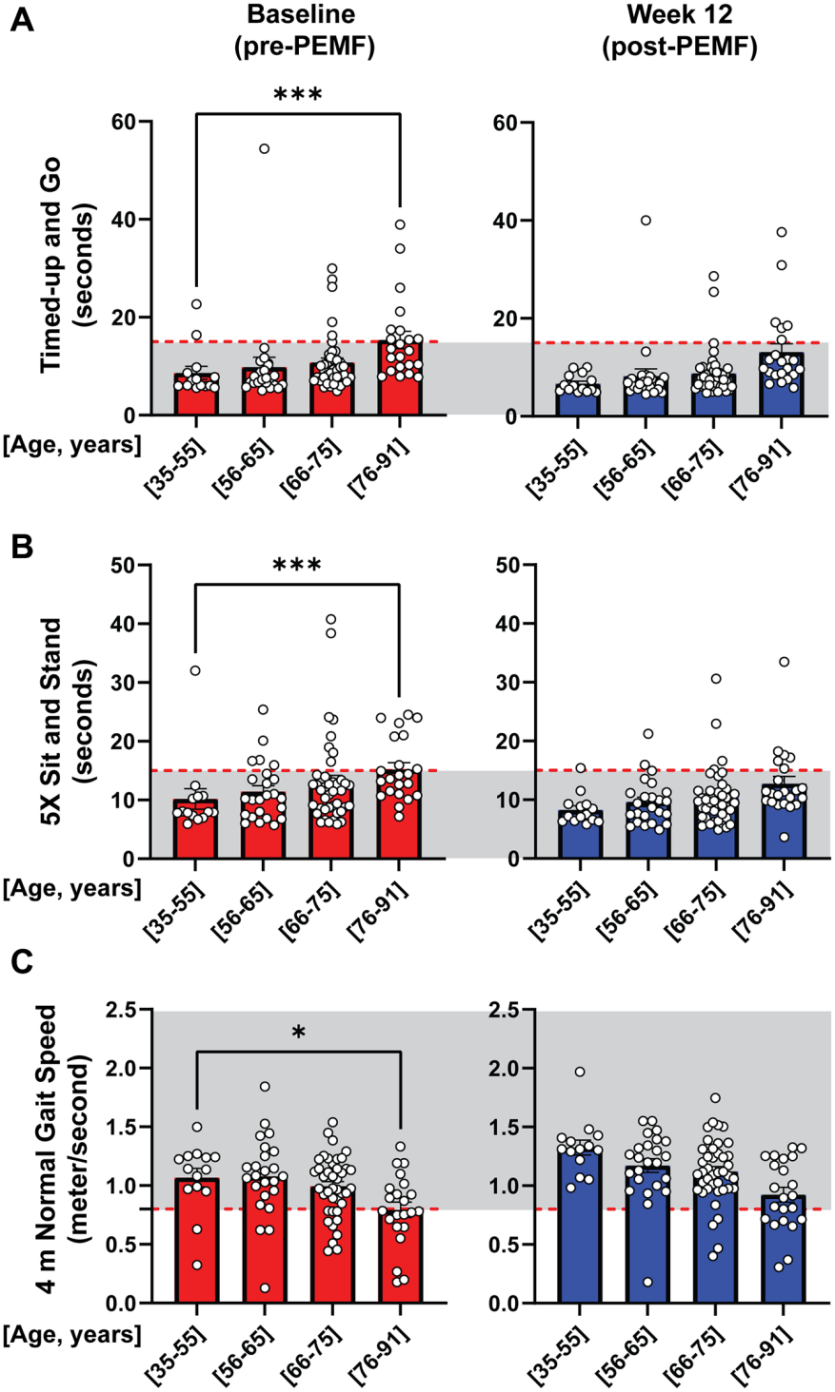
**Age-stratified changes in mobility function, pre- and post-PEMF therapy.** Bar charts depicting age-stratified performance at baseline (pre-PEMF; red bars) and following 12 weeks of PEMF therapy (blue bars) in the TUG (**A**), 5xSTS (**B**), and 4mNGS (**C**) mobility tests. The gray shaded areas represent cutoffs of ≤14 seconds, ≤15 seconds and ≥0.8 m/s indicative of safety from physical failing reported for the TUG [29], 5xSTS [30], and 4mNGS [31], respectively. The number of subjects per age bracket are as follow: (35–55) = 14, (56–65) = 24, (66–75) = 41, and (76–91) = 22. Statistical analysis was carried out using One-Way ANOVA and Kruskal-Wallis multiple comparisons test, with ^*^p < 0.05, ^**^p < 0.01 and ^***^p < 0.001.

Age-associated results for the three functional tests are shown in Figure 2. Sub-group analyses of the functional tests were conducted by stratifying participants into separate age quartiles as depicted in Figure 2A–2C. The red set of histograms reveal statistically significant functional deterioration between 33–55 and 76–91 years of age at study onset (week 0). On the other hand, 12 weeks of PEMF therapy (blue histograms) was correlated with generalized functional improvements across all age groups and tests. Most notably, PEMF therapy (blue histograms) ameliorated age-dependent losses in physical capacity, as demonstrated by a loss of statistically significant differences between the “youngest” (35–55 years of age) and oldest (76–91 years of age) quartiles for all three tests (blue histograms compared with red histograms). PEMF-associated improvements are hence greatest in the older and more frail participants, which are commonly coincident phenotypes.

**Figure 3:**
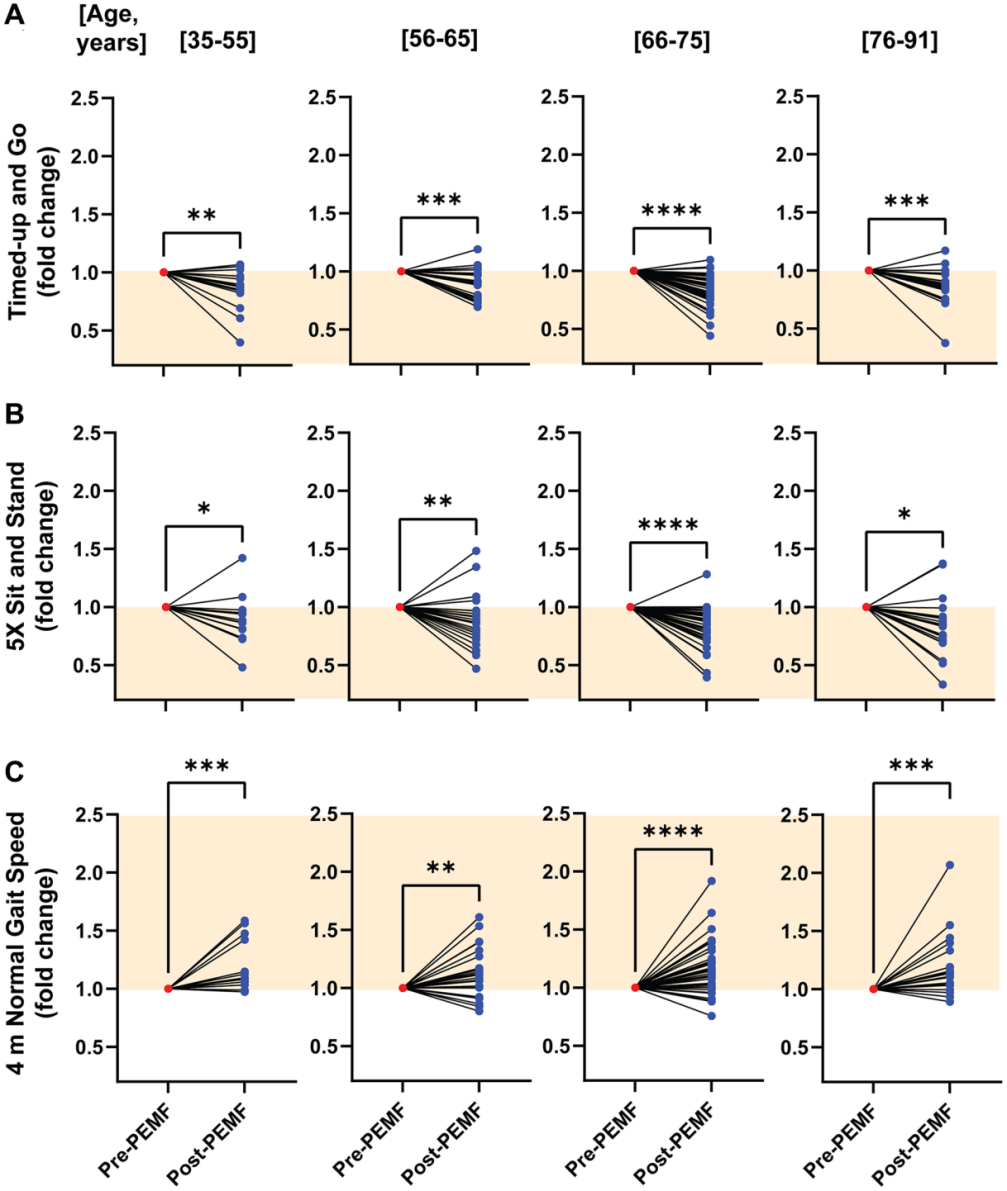
**Normalized age-stratified changes in mobility function before and after PEMF therapy.** Fold change improvements for the (**A**) TUG, (**B**) 5xSTS and (**C**) 4mNGS tests after 12 weeks of PEMF therapy. Data was normalized to the respective baseline score for each subject. The orange-shaded regions depict the direction of improvement in mobility function for each test scenario. Statistical analysis was carried out using the Wilcoxon matched-pairs signed rank test, with ^*^p < 0.05, ^**^p < 0.01, ^***^p < 0.001 and ^****^p < 0.0001. For more information on the total number of responders and non-responders to PEMF therapy, refer to Supplementary Figure 2.

**Figure 4:**
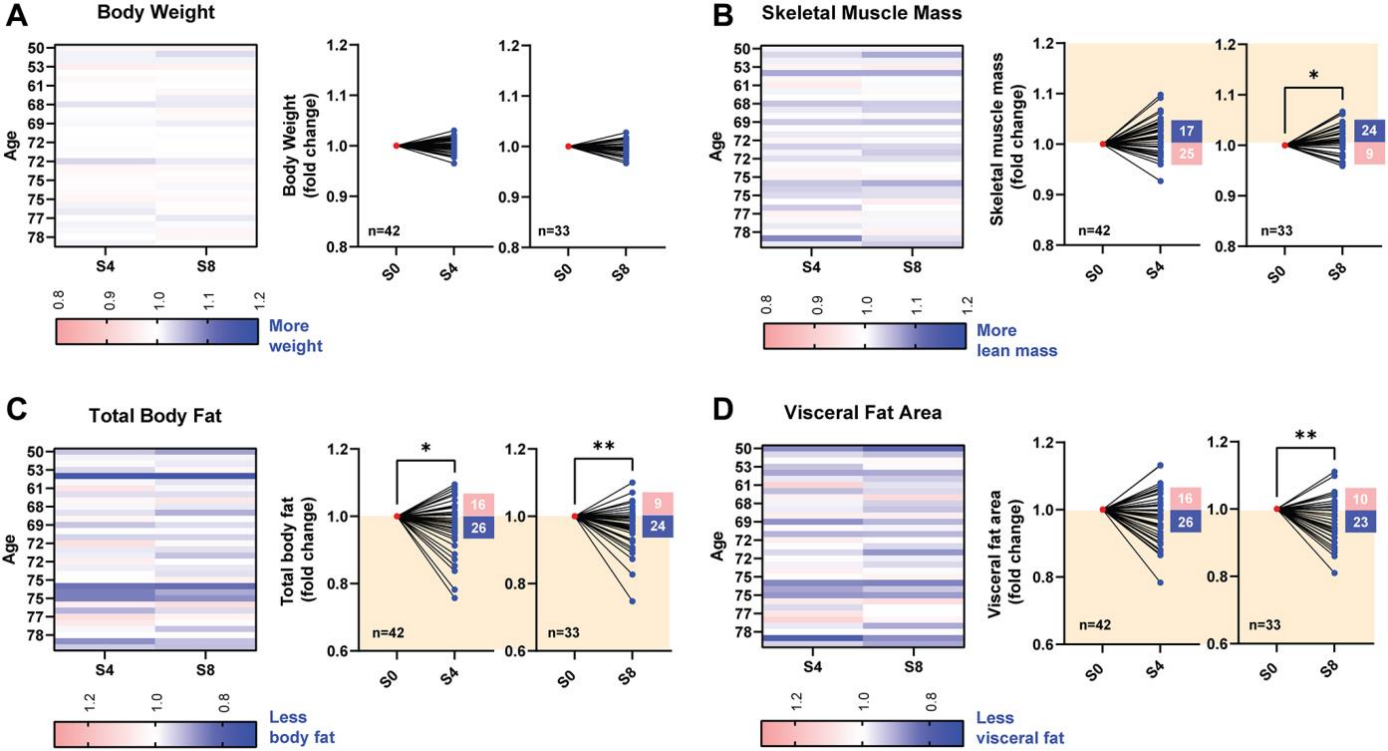
**Changes in body composition before and after 4 and 8 weeks of PEMF therapy.** Body composition assessments were performed using bioelectrical impedance analysis with an InBody device at baseline (pre-PEMF), and after 4 (S4; *n* = 42) and 8 (S8; *n* = 33) sessions of weekly PEMF exposure. (**A**) Changes in body weight, expressed as fold change over baseline measured after 4 and 8 weekly PEMF sessions. Fold changes in skeletal muscle mass (**B**), total body fat (**C**) or visceral fat area (**D**) following PEMF therapy normalized to the respective baseline score for each subject (also see Supplementary Figure 3). The normalized before-after muscle and fat plots depict the fold change over baseline after 4 (S4) and 8 (S8) sessions of weekly PEMF exposure. The orange-shaded regions depict the direction for fold change improvement for each compositional assessment. The number of subjects for each trend direction is indicated in the corresponding box. Statistical analysis was carried out using the Wilcoxon matched-pairs signed rank test, with ^*^p < 0.05 and ^**^p < 0.01.

Supplementary Figure 1 shows raw data for each age group and reveals statistical significance for all three test across all age groups. To more clearly depict group trends, we normalized post- to pre-intervention values for each individual by quartile. Apparent were inter-individual trends towards improvements (orange shaded regions) associated with PEMF therapy that were most significant for the 66–75-year quartile of participants for the TUG (Figure 3A), 5xSTS (Figure 3B) and 4mNGS (Figure 3C) tests. Compared with the different age quartiles, participants in this quartile improved 1.2-fold for both TUG and 5xSTS, and 1.15-fold for 4mNGS. Supplementary Figure 2 provides the “responder” breakdown for each grouping. Notably, the percentage of responders increased after 65 years of age, indicating that the older participants experienced greater gains following PEMF therapy.

Bioelectrical impedance analyses revealed PEMF-associated changes in body composition. Whereas no significant differences in body weight were detected after 8 weeks of PEMF therapy (Figure 4A), a 1.2% (*p* < 0.05) increase in lean muscle mass was observed in ~72% (*n* = 24) of the participants (Figure 4B). By comparison, PEMF-associated changes in total body fat were more pronounced. Significant decreases in total body fat were observed in ~62% (*n* = 26) and ~72% (*n* = 24) of the participants after 4 and 8 weeks of PEMF therapy, respectively, with mean changes in total body fat of ~−3% and ~−4%, respectively (Figure 4C). Significant decreases in visceral fat were observed in ~70% (*n* = 23) of the participants after 8 weeks of PEMF therapy, with a mean change of ~−4% (Figure 4D). Supplementary Figure 3 shows the raw data for each grouping before normalization. Supplementary Figure 4 shows body compositional data for ages between 50–70 and 71–83 and revealed that reductions in visceral fat were greater for the older group after 8 weeks.

**Figure 5:**
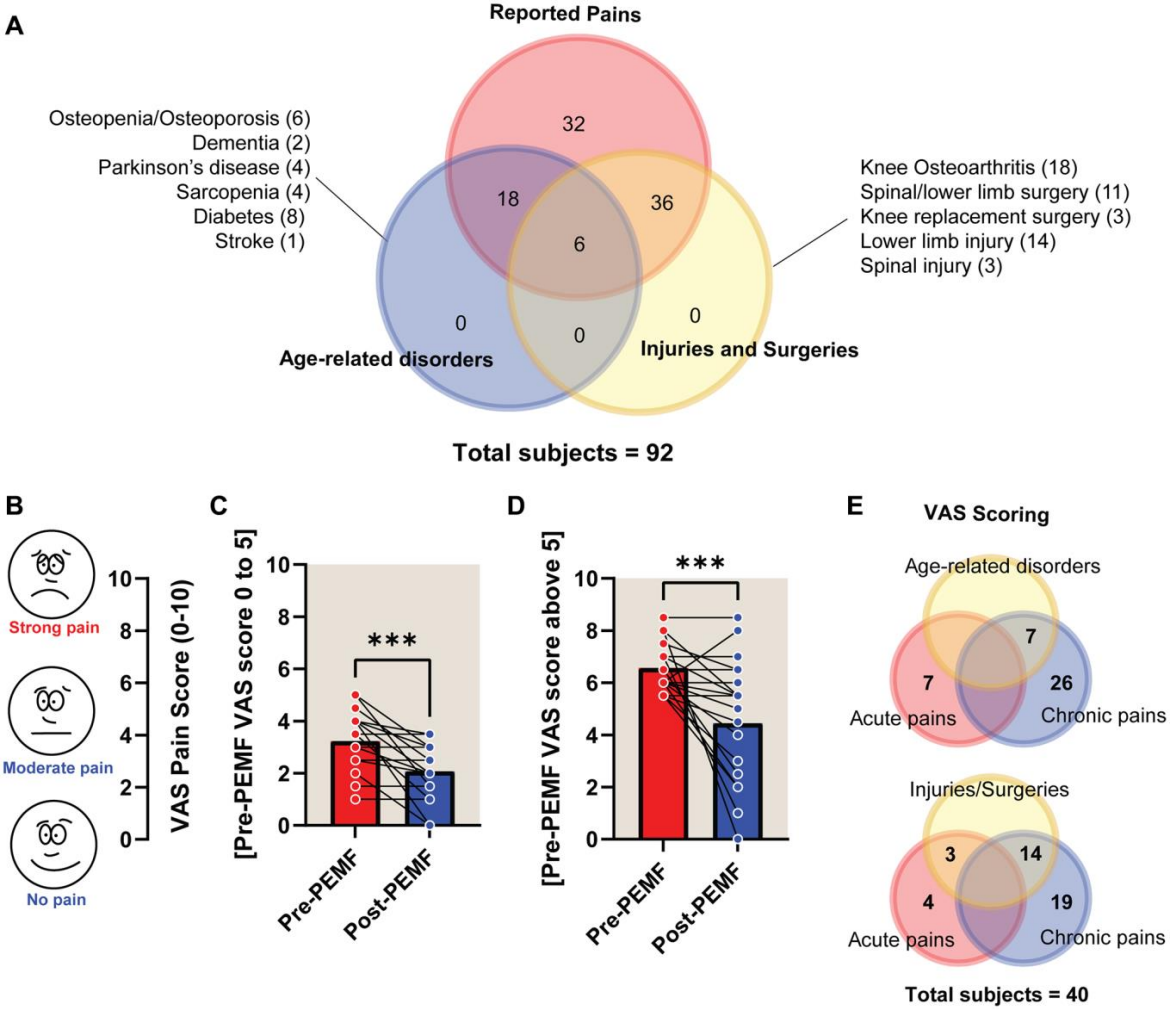
**Visual analogue scale (VAS) scoring of pain pre- and post-PEMF therapy for 12 weeks.** (**A**) Venn diagram depicting the number of subjects (60 females and 32 males) reporting health conditions as either age-related (blue) or arising from injuries and/or surgeries (yellow) and associated pain (red). The number of subjects for each disorder/injury/surgical intervention are indicated within the parentheses. Pain level before and after PEMF therapy was rated using the VAS “faces” pain rating scale (**B**), with a rating of 10 indicating strong pain and a rating of 0 indicating no pain. VAS scores were tabulated based on pre-treatment scores between either (**C**) 0 and 5 (*n* = 19), or (**D**) scores above 5 (*n* = 21). (**E**) 40 of the 92 subjects completed the VAS pain questionnaire, wherein 83% (33/40) reported chronic pain, amongst whom 21% (7/33) reported age-related disorders and/or 42% (14/33) reported past or recent injuries/surgeries. The mean change in VAS score for subjects in the “0 to 5” bracket (**C**) was −1.23 points, from a mean of 3.23 to 2.07, before and after PEMF therapy, respectively. The “above 5” group (**D**) showed a −2.12-point change, from means of 6.57 to 4.45. Statistical analysis was carried out using Wilcoxon matched-pairs signed rank test and showed significant improvement in pain relief with ^***^p < 0.001 after magnetic therapy.

A breakdown of the health conditions of the study participants is shown in Figure 5. Of the 101 study participants (aged 38–91 years; 63 females and 38 males), 92 (60 females and 32 males) reported having pre-existing health conditions, while the remaining 9 did not. Of the 92 participants reporting health conditions, most presented with some degree of pre-existing pain; 72 (78%) were associated with age-related conditions (Figure 5A) and/or 72 (78%) with acute injuries/surgeries (Figure 5B). A Visual Analog Scale (VAS) was used to assess pain before and after PEMF treatment in a subset (40) of the participants (Figure 5B). Significant improvements in low-to-moderate pain perception (VAS:0–5) were reported after PEMF therapy (Figure 5C). Notably, severe pain perception (VAS>5) was more strongly attenuated following PEMF therapy (Figure 5D). Of these respondents, 83% (33/40) reported suffering from some form of chronic pain (Figure 5E). Only one respondent reported a severe worsening of pain following PEMF therapy (Figure 5D).

## DISCUSSION

Muscle’s predominant role in establishing systemic metabolic homeostasis can be attributed to its large and dynamic pool of mitochondria [2, 32]. Succinctly, physical exercise is health- and life-extending because, to be executed, it necessitates the activation of the muscular mitochondrial pool. Mitochondrial respiratory activation and the consequent production of reactive oxygen species (ROS), in turn, serve as triggers to initiate energy-driven enzymatic and transcriptional cascades that ultimately culminate with the activation of the muscle secretome response [33, 34]. The muscle secretome is comprised of a myriad of regenerative, metabolic, anti-inflammatory, and immunity-boosting factors that are released into the systemic circulation either individually [35, 36] or vesicle-encapsulated [37–39]. For the most part, these muscle-derived secreted factors promote the maintenance and metabolism of the muscle itself, as well as that of collateral tissues, thereby accounting for the widely accepted health benefits of exercise. Sarcopenia describes the progressive loss of muscle mass, interdependent with a deterioration of mitochondria function and number, in the older adults and frail. Indeed, evidence exists that accrued mitochondrial dysfunction with advanced age contributes to age-dependent muscle loss via the activation of catabolic pathways [40]. Sarcopenia hence compromises resilience to disease and results in losses of muscle size, strength, and function. Mitochondrial/muscle usage prolongs mitochondrial/muscle functional efficiency hence, these debilitating effects of sarcopenia can be offset to a significant degree by adopting a lifestyle incorporating moderate levels of physical activity and caloric restriction [40]. The systemic metabolic attributes arising from the activation of the muscle mitochondrial pool during exercise are attributed to the enhanced expression and function of the master regulator of mitochondrial biogenesis, PGC-1α. Unfortunately, muscle has been largely overlooked in clinical strategies employing PEMF-based technologies, missing out on a valuable rehabilitative opportunity.

### Sarcopenia: a pathological form of fat-muscle crosstalk

In later life, muscle loss is accompanied by increased ectopic adiposity with grave functional and metabolic consequences. Other than a strong association with age, a problem lies in identifying individuals at greatest risk for early medical intervention. Some investigators have proposed that visceral fat area, instead of Body Mass Index (BMI), should be used as a diagnostic criterion for the disorder when associated with muscle loss [41, 42]. At equivalent BMIs, Asians have been shown to generally possess higher percentages of body fat and to develop metabolic disorders such as type, 2 diabetes and cardiovascular disease, sooner than Caucasians [43, 44]. More specifically in alignment with the present study, Singaporeans have been recently shown to exhibit higher adiposity at comparable BMIs, age, and sex relative to American or European Caucasians [43, 45]. As such, BMI does not adequately reflect the amount of visceral fat or other ectopic atherogenic (inflammatory) adipose deposits, which appear to be determinant and more prominent in the South Asian population, even after adjusting for habitual levels of physical activity [44]. In accordance, the baseline BMIs of the participants of the present study (~23.5 kg/m^2^; Table 3) are considered overweight by the Singaporean Ministry of Health guidelines where a cut-off point of 23 kg/m^2^ has been assigned [43], whereas internationally it is set at 25 kg/m^2^. Notably, only modest, but consistent, drops in BMI were observed across all ages after 8 weeks of PEMF therapy, whereas reductions in total and visceral fat were significant and robust. The fact that once-weekly PEMF exposure (10 minutes) was associated with significant reductions in visceral fat suggests that PEMF therapy holds clinical relevance.

**Table 3:** Mean body mass index (BMI; kg/m^2^) of participants across the different age brackets, after 4-weekly or 8-weekly PEMF therapy.

**4-weekly PEMF**	**(All)**		**(35–55)**		**(56–65)**		**(66–75)**		**(76–91)**	
Number of subjects	42		7		5		18		12	
PEMF Therapy	Pre	Post	Pre	Post	Pre	Post	Pre	Post	Pre	Post
**Weight (kg)**	59.7	59.6	64.3	64.0	51.9	51.7	61.2	61.0	58.4	58.5
Std. Deviation	12.5	12.3	14.0	14.4	3.8	3.4	11.9	11.5	15.6	15.3
**BMI (kg/m^2^)**	23.25	23.21	23.91	23.80	20.88	20.81	23.60	23.53	23.31	23.39
Std. Deviation	4.03	3.99	5.02	5.20	1.66	1.45	3.76	3.64	4.55	4.49
Minimum	17.00	17.31	18.90	18.50	19.50	19.46	17.60	18.13	17.00	17.31
Maximum	33.10	33.21	32.70	32.48	23.30	22.84	33.10	33.21	32.30	32.34
Range	16.10	15.90	13.80	13.98	3.80	3.38	15.50	15.08	15.30	15.03
**8-weekly PEMF**	**(All)**		**(35–55)**		**(56–65)**		**(66–75)**		**(76–91)**	
Number of subjects	33		4		4		17		8	
PEMF Therapy	Pre	Post	Pre	Post	Pre	Post	Pre	Post	Pre	Post
**Weight (kg)**	60.5	60.2	60.5	60.2	52.9	52.5	60.8	60.6	63.6	63.3
Std. Deviation	11.3	11.2	7.5	8.6	3.5	3.6	12.2	11.7	13.2	13.3
**BMI (kg/m^2^)**	23.56	23.44	22.20	22.11	21.08	20.89	23.67	23.57	25.25	25.11
Std. Deviation	3.76	3.67	3.03	3.59	1.85	1.74	3.82	3.61	4.24	4.11
Minimum	17.60	17.88	20.20	19.61	19.50	19.42	17.60	17.88	19.60	19.43
Maximum	33.10	32.64	26.70	27.43	23.30	23.09	33.10	32.64	32.30	31.67
Range	15.50	14.76	6.50	7.82	3.80	3.67	15.50	14.76	12.70	12.24

No significant differences were observed between age brackets at any time of assessment.

Age-related increases in adipose inflammation in combination with decreases in physical activity result in the redistribution of fat to intra-abdominal (visceral fat) and intramuscular sites. The resulting accumulation of intramuscular atherogenic adipose tissue results in the production of reactive lipid species (lipotoxicity), mitochondrial dysfunction, and oxidative stress, that depress β-oxidation of fatty acids, promote insulin resistance, stimulate the secretion of pro-inflammatory cytokines, a cascade of events that generally undermine muscle viability. This shift in the muscle secretome towards pro-inflammation further exacerbates adipose inflammation, leading to a state of system-wide inflammation and the establishment of a vicious cycle of metabolic and functional decline that define the pathogenesis of sarcopenia [41, 46]. The resultant inflammatory systemic milieu is also hyper-responsive to even minor systemic perturbations, such as infections, that plays a major role in the pathophysiology of frailty [47]. Therefore, the appropriate compositional balance between muscle and fat, particularly visceral and intramuscular fat, is of utmost importance in maintaining healthspan through the modulation of body energy efficiency, myokine-adipokine crosstalk and systemic inflammatory status. Here, we report improved maintenance of skeletal muscle in conjunction with reductions of total and visceral adipose that moreover, were accompanied by improvements in indices of functional mobility, most significantly in the elderly. PEMF-based technologies may hence represent a valuable adjuvant therapy to conventional geriatric interventions intended to reduce the prevalence of frailty in the older adult population.

### Muscle-targeted PEMF therapy and metabolic stabilization

In recent years the cellular responses mobilized by PEMF exposure have come into clearer focus and commonly impinge upon calcium signaling and mitochondrial respiration (ROS) to reach fruition [14, 20, 48–53]. One calcium-permeable channel in particular, the Transient Receptor Potential Cation Channel Subfamily C Member 1, or TRPC1, has received attention [20, 22, 23, 27, 28, 52, 54, 55]. A TRPC1-mitochondrial axis has been revealed that can be induced by magnetic stimulation and hence possesses the necessary functionality (calcium entry and mitochondrial interaction) to coalesce both aspects of the noted response cascade [22, 55]. Notably, exercise-dependent activation of TRPC1-mediated calcium entry promotes the development of oxidative muscle that is necessary for the execution of physical activities requiring endurance and in maintaining normal posture, and thus confers resistance to fatiguing physical activities [56–58]. Oxidative muscles are major contributors to systemic metabolic flexibility [59] and exhibit a predilection for fatty acid oxidation [60], traits that are related to their elevated mitochondrial content [61]. Accordingly, oxidative muscle was shown to be particularly responsive to magnetic exposure. For instance, 5 weeks of PEMF treatment (10 minutes/week) was sufficient to improve the running performance of mice compared to unexposed littermates [24]. The production of mitochondrial ROS and subsequent activation of PGC-1α transcriptional cascades are responses shared by both exercise [2, 32, 60–62] and our magnetic paradigm [22, 23]. The same magnetic stimulation paradigm used in the present study, consisting of brief exposures (10 minutes) to extremely low frequency (Hz-100 Hz) and low amplitude (1.5 mT) PEMFs was previously shown to stimulate both *in vitro* [22] and *in vivo* [24] myogeneses towards the oxidative phenotype, and to emulate the molecular signals characteristic of the metabolic and mitochondrial improvements associated with exercise in isolated muscle cells [22], mice [24] and humans [25]. Given this experimental backdrop, it is not entirely unexpected that this magnetic paradigm should improve functional performance and body composition in an older cohort as demonstrated in this report.

The age stratification of the standard mobility function tests reported here reveals an age-dependent decline in mobility and functional capacity at the commencement of the study (pre-PEMF intervention), trends that agree with a previous study examining the spontaneous decline in mobility function using the International Classification of Functioning, Disability and Health (ICF) framework [63]. This recent study reported a significant deterioration in the physical functioning of older adults (60–90 years) after one year of monitoring, demonstrating an increase in time taken for the TUG test from a mean of 12.8 to 14.5 seconds (*P* < 0.001), a decrease in the sit-stand 30-second chair stand test from a mean of 10 to 8 repetitions (*P* = 0.001), and an increase in the time taken for the 10-minute walk test from a mean of 12.4 to 14.4 seconds *(P* = 0.001) [63]. In an inverse manner, our study demonstrated improved mobility function using a similar set of functional tests (TUG, 5xSTS and 4mNGS) following PEMF therapy. With reference to the TUG test used by both studies, we demonstrated reductions in mean time scores in older subjects (>65 years), of greater absolute magnitude, but in the opposite direction (improvement, instead of worsening) and over a shorter span of time (3 months versus one year) (Table 2) associated with weekly PEMF intervention. The pre-intervention baseline values for the TUG in the present Singaporean study (15.35 seconds, 76–91 years of age) and post-assessment baseline values in the Turkish study [63] (14.5 seconds, 60-90 years of age), however, were of similar magnitude. Notably, an increase in the amount of time taken for the TUG test is associated with increased risks of falls, fractures, cardiovascular disease, dementia and Parkinson’s disease [64–68]. Longer TUG scores are also correlated with increases in fat mass, but not BMI, in an exclusively Singaporean cohort [45]. Therefore, our observation that PEMF therapy was associated with both improvements in TUG score and reduced adiposity but had less of an association with BMI (Table 3), aligns with previous studies and moreover, suggests that PEMF therapy holds potential therapeutic value for the older adult population in the areas of cognitive and physical decline, including reducing the risks of falls.

Improvements in exercise performance, adipose browning and fatty acid oxidation have been reported in small animal studies employing an analogous PEMF platform [24]. Notably, the improvements we detected in this human cohort are comparable to those previously reported for exercise intervention in the older adult population. Employing dual x-ray absorptiometry (DEXA) measurements, Aas et al. (2020) [69] detected a 1.5% increase in lean leg mass after 10 weeks of thrice weekly resistance exercise and protein supplementation in an elderly cohort (~85 years), compared to a 1.2% increase in lean body mass reported here employing bioelectrical impedance analysis after 8 weeks of PEMF therapy. For the 71–83 age group specifically, a 1.1% increase in lean body mass was detected after 8 weeks (Supplementary Figure 4). In a systematic review and meta-analysis, Lu et al. (2021) [70] reported improvements in TUG times of −0.66 seconds (standardized mean differences across studies) in the elderly (>60 years) in response to exercise training and further asserted that the TUG is a better predictor of exercise adaptations than other mobility tests. For comparison, our eldest age quartile (76–91) exhibited the greatest improvements in mean TUG time of −2.21 seconds (Table 2). Notwithstanding differences in methodologies of assessment, these studies suggest that PEMF therapy can produce comparable results to exercise in older individuals.

### Magnetic mitohormesis: not a paradox, but an opportunity

Mitochondria can be considered the cell’s environmental stress sensors of a manner that is biologically adaptive [26]. Mitohormesis refers to an adaptive process whereby low levels of oxidative stress confer the installation of survival adaptations and promote regeneration, whereas greater levels of oxidative stress can stymie cell growth and survival [71]. As magnetic fields stimulate mitochondrial respiration, they can be exploited as a method with which to non-invasively produce mitohormetic responses, via a process of Magnetic Mitohormesis. Traditionally, the selection of magnetic exposure regimes has not been made with mitohormesis taken into mechanistic consideration, ultimately giving rise to disparate findings between analogous preparations. On the other hand, if taken into consideration, exposure regimes can be potentially designed to target a specific objective. For instance, we have recently shown using the same magnetic technology that stronger magnetic stimulation can be used to specifically halt breast cancer growth, employing the same molecular machinery [72]. Therefore, PEMF-based therapeutic strategies can be ultimately designed to either promote or arrest development depending on exposure intensity (duration, amplitude, and frequency) and the inflammatory status of the tissue in question. Mitohormetic principles must hence be taken into serious consideration to appropriately design efficacious PEMF-based clinical therapies.

### Study limitations

The main caveat to this study was that the participant base consisted of walk-in volunteers of heterogeneous characteristics at the commencement of the trial. Notwithstanding, measured improvements were stronger in the older participants, which goes against the accepted trend of worsening with advanced age [63], as corroborated in the present study. Further suggesting an authentic therapeutic effect, improvements were consistently observed across diverse measures including the perception of pain, functional mobility, as well as body composition. Changes in body composition, such as visceral adiposity, can be considered as objective measures and less subject to perceptual or psychosomatic bias. Visceral fat content was recently shown to be less responsive to physical activity in the South Asian population [63], yet was reduced following PEMF therapy in the present Southeast Asian study, also arguing for therapeutic specificity. Given that age-related increases in visceral adiposity are a major contributor to the pathogenesis of sarcopenia [42] and its reported persistence in the Southeast Asian population [43, 44], the observation that our PEMF paradigm produced the greatest amelioration of visceral fat in our Southeast Asian older cohort (Supplementary Figure 4) indicates that our magnetic intervention holds both functional and metabolic contributions that are relevant for human aging and frailty. The possibility that the observed effects can be attributed to physical improvement with time also seems unlikely since it would be in opposition to general trends in this cohort and elsewhere [63] to naturally worsen with age. There was also no restriction on other rehabilitative measures imposed by the study team. Nonetheless, of those reported, outside rehabilitation had no effect on any of the functional assessment scored (Supplementary Figure 5), suggesting that the observed improvements were for the most part attributed to the PEMF intervention. Finally, there was no imposed restriction on pain medications during the study period which could have contributed to the reported pain reduction after PEMF therapy. However, most of the reported pain (~83%) was chronic in nature and putatively had been unresponsive to various analgesics. Although a placebo effect cannot be totally ruled out as having had an effect in the results reported for this case study, a primary placebo contribution is unlikely due to the consistency, breadth and nature of the responses observed following PEMF treatment. Moreover, the measured improvements in mobility function and body composition associated with PEMF therapy met and surpassed the cut-offs previously established in published studies [29–31, 63–68, 70] that would have needed to be exceeded in order to offset human age-related frailty and which generally worsens with age, particularly in the older adult population, where we detect greatest improvements. Therefore, the sum of these results implies an authentic therapeutic effect can be attributed to PEMF therapy. Given the potential clinical relevance of our magnetic field paradigm, further clinical investigation is certainly warranted.

## CONCLUSIONS

Therapies targeting skeletal muscle hold major advantages over other tissues given muscle’s broad systemic ramifications. Here we provide initial findings that brief weekly PEMF exposure of the upper limb of humans produces clinically relevant improvements in pain, mobility, and indications of lean muscle mass, in an age-dependent manner. In accordance with previously published animal preclinical [24] and human clinical [25] studies employing analogous PEMF paradigms, adipose tissue homeostasis was particularly responsive to PEMF intervention. Most notably, the older subjects exhibited the most significant improvements in mobility and body composition. Although the results reported here are very promising, they remain to be substantiated and broadened in randomized controlled clinical trials.

## METHODS

### Subject recruitment

Male and female voluntary subjects without any surgical implants, who were not pregnant, without any major mobility issues, and over the age of 21 years were allowed to participate in the PEMF therapy program at the QuantumTx Demo Centre. To be included in this retrospective study, only subjects above the age of 35 years were considered and must have completed the 12-week weekly PEMF therapy with complete data points. Most study participants were word-of-mouth referrals from previous clients or joined the programme after seeing press releases of the technology. Informed consent was taken before the start of the program. This retrospective study was conducted as per the Declaration of Helsinki and approved by the NUS Institutional Review Board (NUS-IRB-2022-841).

### PEMF exposure

The PEMF paradigm employed here for use in human clinical trials has previously been described [25]. The quadriceps region of subjects was exposed to PEMFs (1 mT) once a week for 10 min, over 12 weeks, on alternating legs each week. All participants were required to undertake 12 sessions of PEMF exposure. Approximately 85% of the study participants completed 12 weekly PEMF sessions without interruption; full compliance. Due to holidays, travel or illness, however, the remaining subjects completed 12 PEMF sessions within 16 weeks.

### Functional and mobility assessments

Each participant completed a series of standard performance-based functional tests with modification [64–68, 73] at baseline (Week 1) and the end of the program (Week 12), to assess improvements in mobility function. These tests include (1) Timed Up and Go Test (TUG), (2) Five Times Sit to Stand Test (5xSTS), and (3) a 4-meter Normal Gait Speed (4mNGS). For TUG, seated participants were timed (in secs) from the start of rising from a chair and returning to a seated position after completing a walk and turnaround of 3 meters. For 5xSTS, seated participants were assessed for the time (in secs) required to complete a stand and sit 5 times with arms crossed against their chest. The 4mNGS test records the duration (in secs) required of the participants to complete a 4-meter walk at a comfortable pace.

### Pain assessments

A Visual Analog Scale (VAS) was used for pain assessment and has been shown quite reproducible in accurately assessing chronic pain levels [74]. For the Visual Analog Scale (VAS) pain score, participants indicated their existing acute and chronic pains based on the VAS “Faces” pain rating scale from 1 (no pain) to 10 (severe pain), at week 1 and week 12.

### Body compositional assessments

The measurement of the subject’s body composition was conducted using the Inbody™ 770 device (Inbody Co., Ltd.) before each weekly PEMF session and data parameters such as weight, skeletal muscle mass, body fat mass, and visceral fat area were collected at after session 4 and session 8. Body Mass Index (BMI) was calculated by dividing the weight (kg) of a person by the square of height (m^2^) and was an output measure of the Inbody™ 770 device.

### Statistical analysis

Given that the raw data did not pass normality tests, non-parametric test was used. The Wilcoxon matched-pairs signed rank test was carried out to compare pre-and post-PEMF therapy on raw data and transformed data. Transformed data were expressed as fold change over the baseline (week 1) of each subject. The One-way ANOVA followed by Kruskal-Wallis tests was carried out to analyse the statistics for multiple comparisons. All statistical analyses were performed using GraphPad Prism version 9 for Windows.

### Data availability statement

The original anonymous dataset is available on request from the corresponding authors.

## Supplementary Materials

Supplementary Figures
