# Supplementary material for: Brief, weekly magnetic muscle therapy improves mobility and lean body mass in older adults: a Southeast Asia community case study
Source: Aging (Albany NY). 2023 Mar 19;15(6):1768–90. doi: 10.18632/aging.204597 (PMC10085623; doi:10.18632/aging.204597)
Supplement: Supplementary Figures [file aging-15-204597-s001.pdf]

SUPPLEMENTARY FIGURES

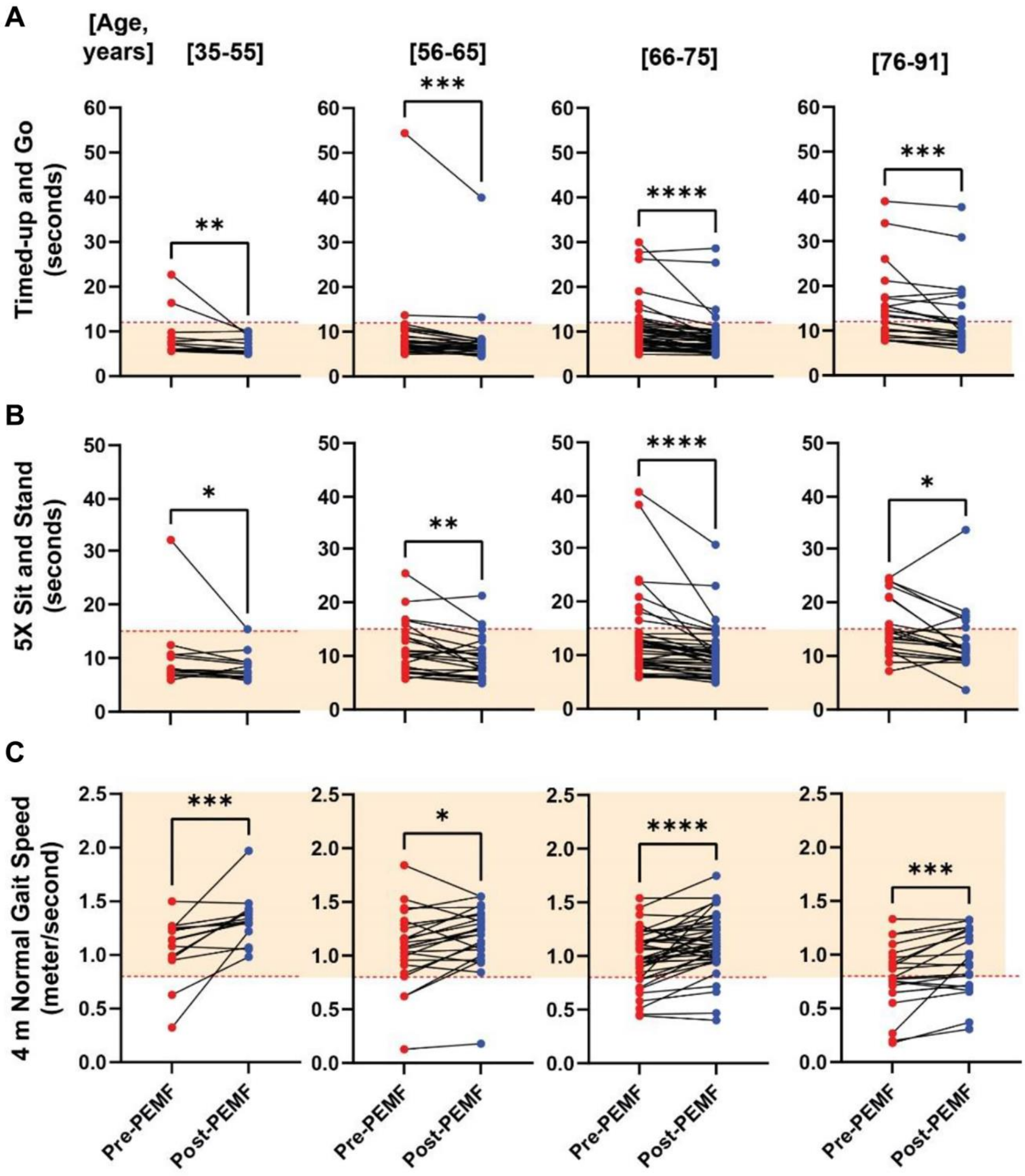

Supplementary Figure 1. Raw data before-after plots for functional assessments. (**A**) Timed-up and Go (seconds), (**B**) 5X Sit and Stand (seconds), and (**C**) 4 m Normal Gait Speed (meter/second). Statistical analysis was carried out using Wilcoxon matched-pairs signed rank test, with $^{*}p < 0.05$, $^{**}p < 0.01$, $^{***}p < 0.001$ and $^{****}p < 0.0001$. The orange shaded regions represent the favorable cutoff areas for each mobility test.

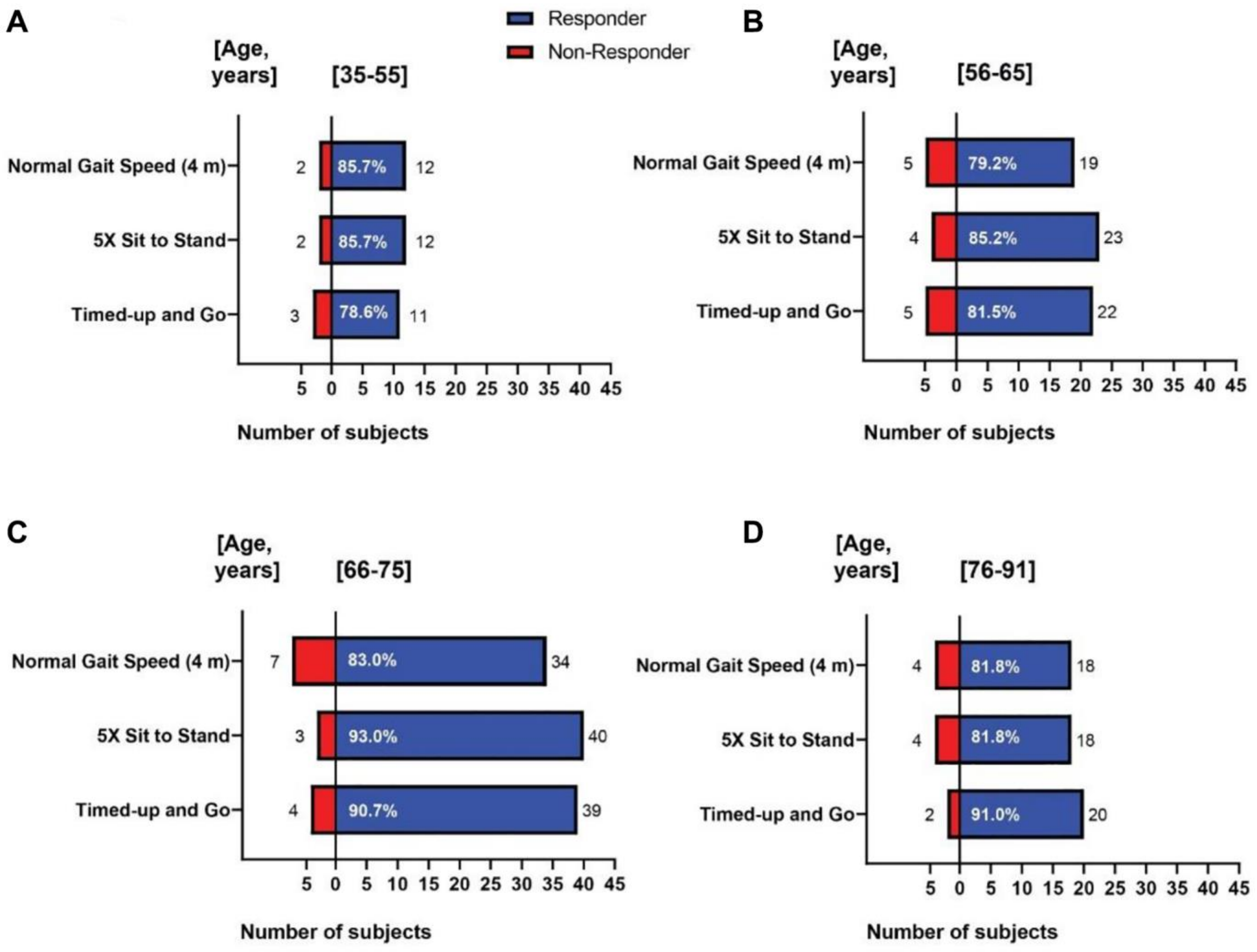

Supplementary Figure 2. Responder histogram of functional mobility tests according to age group for (**A**) years [35–55], (**B**) years [56–65], (**C**) years [65–74] and (**D**) years [76–91]. Greater number of responders in each category. The number of non-responders is more consistent.

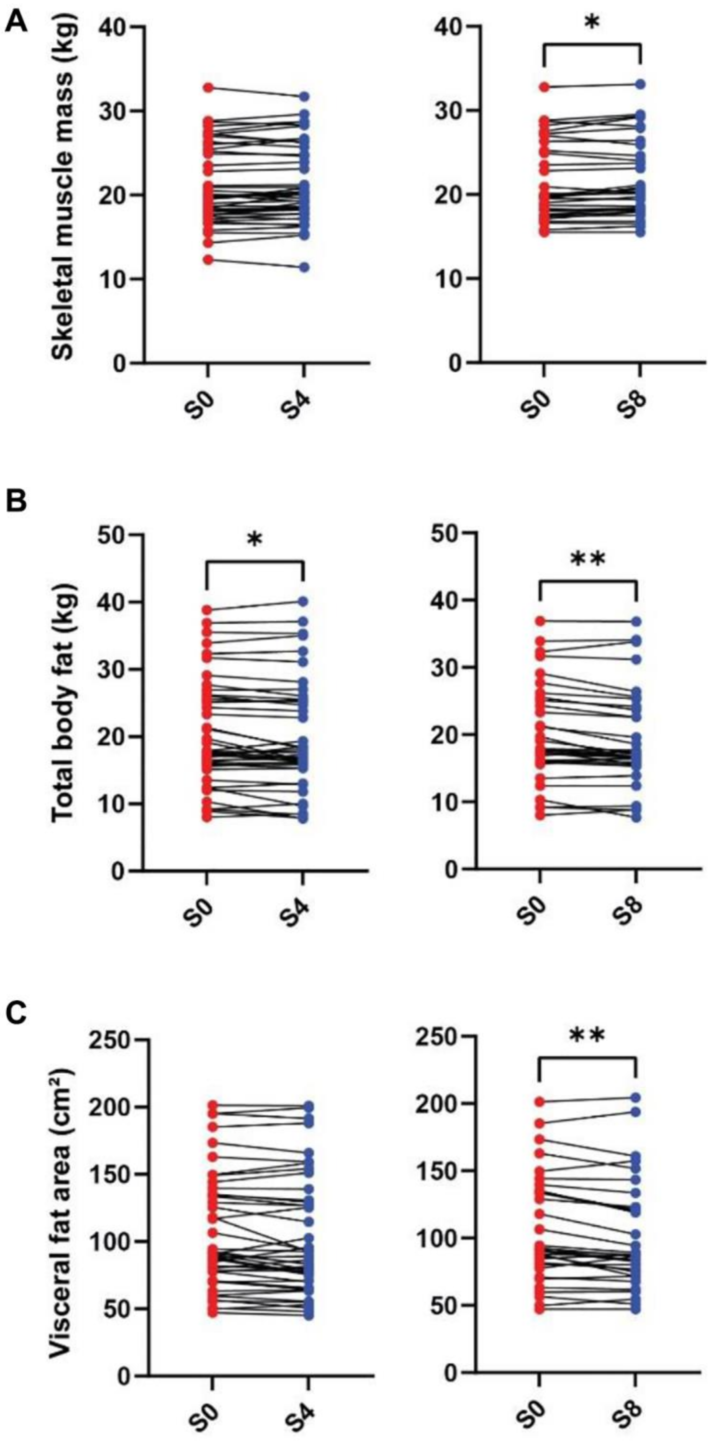

Supplementary Figure 3. Raw data before-after plots for compositional assessments. (**A**) Skeletal muscle mass (kg), (**B**) total body fat (kg) and (**C**) visceral fat area (cm^2). Statistical analysis was carried out using Wilcoxon matched-pairs signed rank test, with $^{*}p < 0.05$, and $^{**}p < 0.01$.

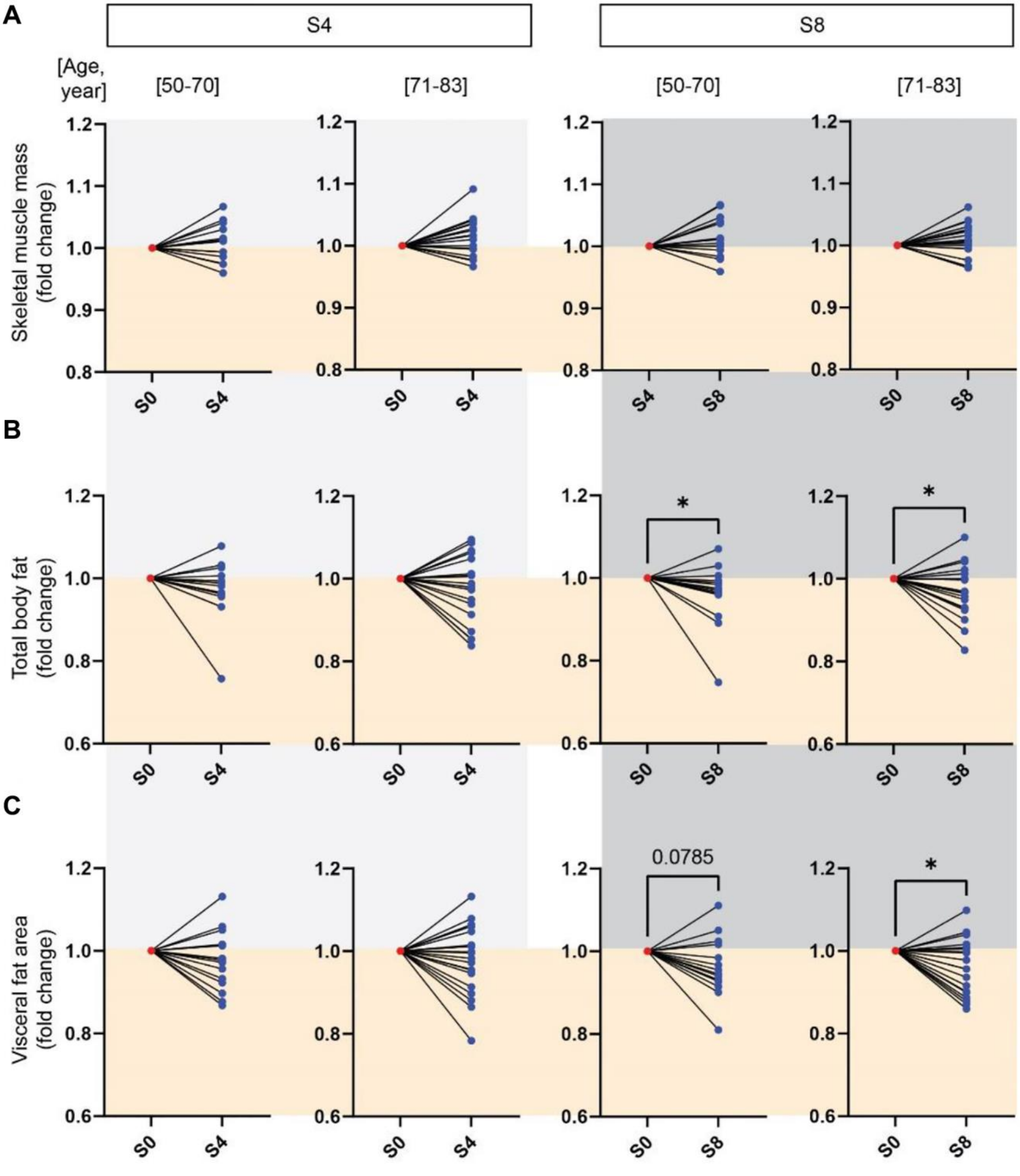

Supplementary Figure 4. Fold change before-after plots for compositional assessments. Data were stratified according to age brackets (50-70 years) and (71-83 years) after 4 (S4) or 8 (S8) magnetic therapy sessions. (**A**) Skeletal muscle mass, (**B**) total body fat and (**C**) visceral fat area. The orange shaded regions represent the favorable fold change improvement for each mobility test. Statistical analysis was carried out using Wilcoxon matched-pairs signed rank test, with $^{*}p < 0.05$.

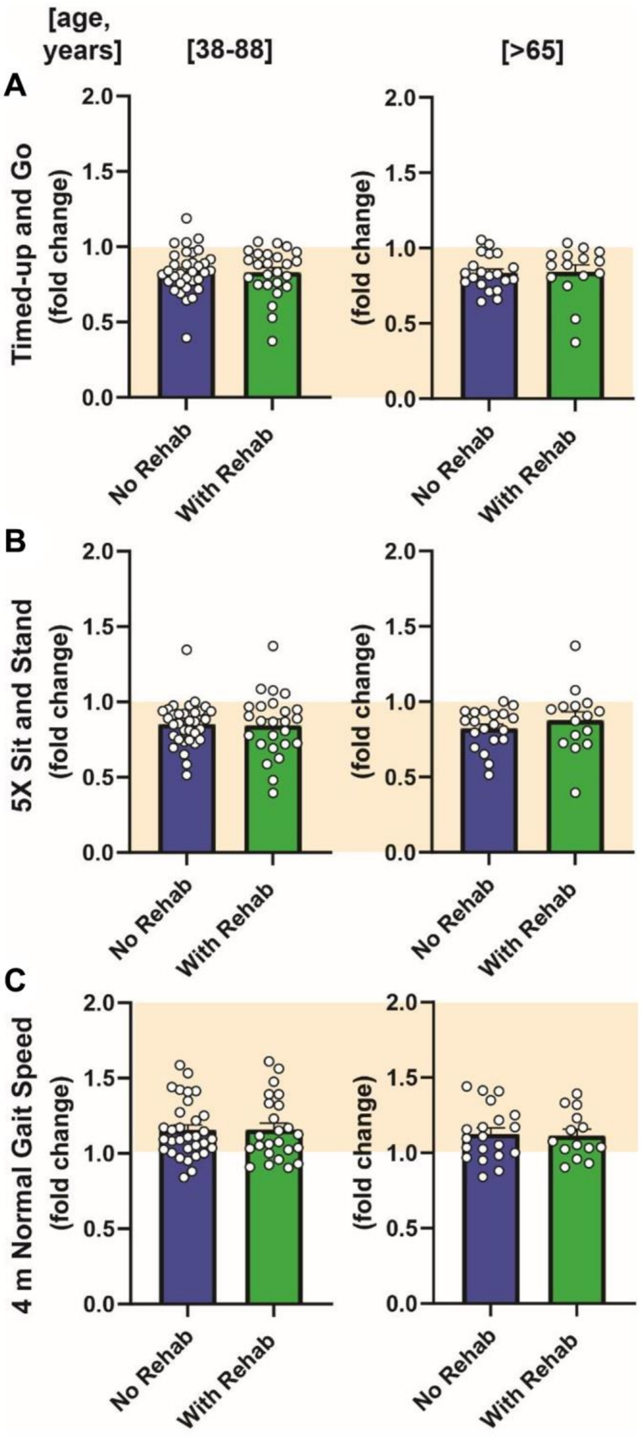

Supplementary Figure 5. No significant difference in the mean fold change improvement for (**A**) TUG, (**B**) 5xSTS and (**C**) 4mNGS of subjects with and without rehabilitation (Rehab) after 12 weeks of PEMF therapy. The assessment scores were tabulated based on subject's self-declared information on rehabilitation activities outside PEMF therapy or not. Reported rehabilitation activities include physiotherapy, chiropractic treatment, and Traditional Chinese Medicine (TCM) treatment such as cupping and acupuncture, done once a week to once a month for the duration of the 12 weeks. The age bracket (in years) and the number of participants for No Rehab and With Rehab are as follows: [38–88] No Rehab = 33, [38–88] With Rehab = 25, (>65) No Rehab = 20 and (>65) With Rehab = 15.
